# The Impact of Unhealthy Behaviors on Personalized Well-Being Index in a Sample of School Dropout Adolescents

**DOI:** 10.3390/children9081144

**Published:** 2022-07-29

**Authors:** Maria Francesca Lodovica Lazzeri, Francesca Mastorci, Paolo Piaggi, Cristina Doveri, Anselmo Casu, Gabriele Trivellini, Irene Marinaro, Andrea Bardelli, Alessandro Pingitore

**Affiliations:** 1 Clinical Physiology Institute, CNR, Via Moruzzi, 56124 Pisa, Italy; mariafrancesca.lazzeri@ifc.cnr.it (M.F.L.L.); cristina.doveri@ifc.cnr.it (C.D.); webmaster@easywebmaster.eu (A.C.); gabriele.trivellini@ifc.cnr.it (G.T.); irene@ifc.cnr.it (I.M.); andreab.bardelli@gmail.com (A.B.); pingi@ifc.cnr.it (A.P.); 2 Department of Information Engineering, University of Pisa, 56122 Pisa, Italy; paolo.piaggi@gmail.com

**Keywords:** health-related quality of life, risk factors, school dropout, adolescence, eating disorder, social context, emotional status, lifestyle habits, psychotropic drugs

## Abstract

(1) Background: here is a growing need for integrated and multidimensional approaches to health, especially in a particular category of populations, school-dropout (SD) adolescents, who are traditionally more prone to risky behavior. This study aimed to describe the association between possible risk factors (substance use, eating disorders, social addiction) and well-being perception through the application of a personalized well-being index (PWBI) in SD youths. (2) Methods: Data were collected in 450 school-dropout adolescents (19 ± 2 years, male 308); the health-related quality of life (HRQoL) and risk behaviors were assessed by means of a battery of standardized questions. (3) Results: The results revealed an altered perception of well-being in association with eating disorders (*p* < 0.001), the use of psychotropic drugs (*p* < 0.001), and the amount of their consumption (*p* < 0.05). In particular, there was a decrease in emotional state (*p* < 0.001) and PWBI (*p* < 0.001) in the presence of eating disorders, and an impairment in all PWBI components, emotional states (*p* < 0.001), lifestyle habits (*p* < 0.05), and social contexts (*p* < 0.001) when taking psychotropic drugs. (4) Conclusions: risk or unhealthy behaviors significantly worsen individual well-being. This study highlights the change of paradigm from a disease-oriented model to an educationally strength-based model when monitoring psychosocial well-being in order to define preventive and health promotion strategies in a vulnerable category of the population.

## 1. Introduction

Adolescence is usually considered to be a time of contradictions. It is both the healthiest period of a lifespan in terms of measurable parameters of psychophysical health, and the age in which risk factors can have crucial effects, predisposing one to chronic diseases later in life. This dichotomy is mainly due to a combination of genetic, physiological, environmental, and behavioral dimensions correlated to a neurobiological phenomenon, called “synaptic pruning”, which may also explain adolescents’ attraction to the discovery of new emotions and pleasures [1]. The combination of this biological vulnerability and several negative events such as parental loss, chronic stress, social isolation, or low quality of life increases the susceptibility to engage in risky behaviors such as the consumption of alcohol and other substances of abuse, tobacco, internet and social media use/abuse, and unprotected sex [2,3,4]. These unhealthy behaviors can sometimes impair social relationships and be responsible for emotional instability, which in many cases leads adolescents to turn to specialists and initiate supportive drug therapy. Recent data, for example, collected in Germany, show a worrying increase in prescriptions for stimulants, antidepressants, and antipsychotics in children and adolescents [5]. This instability, which has a negative impact on the psychosocial dimension, could be responsible for a growing phenomenon, school dropout, understood as the process of leaving school before attaining a minimal qualification [6]. In fact, although it is often tricky to differentiate causes from consequences, it is likely that school dropout is due to the changes that occur during adolescence in physical, cognitive, social, and emotional dimensions, with a particular focus on internalizing disorders such as anxiety and depression [7]. In addition to this age-related vulnerability, which is amplified by school dropouts that appear to correlate with a low health status in adult age, growing evidence indicates that adolescence is also a dynamic and flexible period of knowledge and adaptation to targeted health interventions, so that adolescents can make positive lifestyle choices to enhance their well-being [8]. However, to our knowledge, in order to define new strategies for school dropout prevention and treatment, there is no available framework for psychosocial monitoring that encompasses different dimensions of health, including lifestyle habits, emotional status, social context, and risk behaviors. Therefore, the aim of this study was to assess the Personalized Well-Being Index (PWBI), previously developed as part of the project “A new purpose for promotion and eVAluation of healTh and well-being Among healthy teenageRs” (AVATAR), in a sample of school dropout adolescents to evaluate if any risk behaviors are capable of modifying the perception of well-being in this study population [9]. 

## 2. Materials and Methods

### 2.1. Study Population

Data from school dropout were collected in October 2019 as part of the AVATAR project. AVATAR web-tool aims to develop a platform to evaluate different aspects of dayly life, in particular lifestyle habits, social context, emotional status, and school performance in all phases of adolescence, and to develop an integrated index of the best indicators of well-being in order to quantify the role of variables in the definition of well-being [10]. A total of 680 boys and girls, aged between 18 and 21 years, were included. School dropout adolescents were referred to the Tuscany Region’s FORMATICA Agency for a two-year professional training course. This agency deals precisely with recovering those adolescents who have voluntarily interrupted their studies, offering them a training opportunity for a better integration into the professional world.

Of the 680 adolescents, 230 were excluded according to the inclusion criteria: diagnosed with neuropsychiatric or other diseases (*n* = 20), absence of signature of informed consent (*n* = 75), or questionnaires not filled out completely (*n* = 135). Therefore, the closing population consisted of 450 adolescents (19 ± 2 years, males *n* = 308). School-dropout adolescents were previously trained on how to fill out the questionnaires and conduct the tests. All tests were completed during participants’ computer lesson during professional course. No incentives were provided to adolescents or parents. A practitioner was available to provide information and technical support to complete questionnaires. 

### 2.2. Ethical Considerations

The research protocol was approved by the local ethics committee review board on 15 February 2021. All patients or legal guardians gave informed consent and authorized physicians to use their clinical data in accordance with the Italian law. All procedures performed in the study were in accordance with the ethical standards of the institutional and/or national research committee and with the 1964 Helsinki declaration and its later amendments or comparable ethical standards (Regional Ethics Committee for Clinical Trials of the Tuscany Region 36/2021). 

### 2.3. Procedure and Measurements

Data were collected with AVATAR Web-tool [10]. A socio-demographic data record was used to collect information on gender, age, and schooling. The Italian version of KIDSCREEN-52 was used to assess the health-related quality of life [11,12]. The KIDSCREEN is a self-report questionnaire designed to address the health-related quality of life (HRQoL), aimed at monitoring and measuring the personal experiences of children and adolescents regarding their perception of health status and well-being. The questionnaire, which describes physical, psychological, mental, social, and functional aspects of well-being, consists of 52 items grouped in 10 dimensions [11,12]. The KIDSCREEN questionnaire was psychometrically tested using data obtained in a multicenter European study that included a sample of 22,827 children recruited from 13 countries [13]. Substance use and abuse (nicotine, cannabis, psychotropic drugs, cocaine, heroin, inhalants, amphetamines, alcoholic drinks) was assessed as being present if participant reported having used substances one or more times per week. Experiences in the use of social media over the past year were evaluated with the Bergen Social Media Addiction Scale (BSMAS) [14], containing six items reflecting the core addiction elements (i.e., salience, mood modification, tolerance, withdrawal, conflict, and relapse). Eating attitudes and behaviors were assessed using EAT-26, consisting of three subscales: Dieting, eating preoccupation, and oral control [15]. 

### 2.4. AVATAR Approach: Psychological Well-Being Index

The AVATAR approach consists in focusing on the integration of three components of health-related well-being (lifestyle habits (LH); emotional status (ES); and social context (SC)), as perceived by adolescents [16]. The three components were obtained from the different variables analyzed by the questionnaires according to a structural model previously described in Mastorci and colleagues [9].

In detail, the path analysis technique used measures the extent to which the model fits a dataset and allows for the testing of the interrelationships among several variables simultaneously. Confirmatory factor analysis was used to test an overall measurement model that included five correlated latent variables. The overall model fit was assessed using different statistics. First, a chi-square analysis was used. The other indices were the root mean square error of approximation (RMSEA) (values between 0.05 and 0.08 indicate an acceptable fit, and values <0.05 indicate a good fit), the comparative fit index (CFI) (values >0.90 indicate a reasonable fit, those >0.95 indicate a good fit), and the standardized root mean square residual (SRMR) (values <0.10 indicate a good fit). The measurement model was first tested to ensure that each of the observed variables was a sufficient indicator of the hypothesized latent variables.

From the sum of the three components, we obtained a personalized well-being index (PWBI), ranging from 0 to 100, according to the AVATAR model as reported before [9]. 

### 2.5. Statistical Analysis

Statistical data analyses were performed using SPSS software. Data are presented as mean  ±  SD. The Saphiro–Wilk test was used to confirm the normality of the data distribution for continuous variables before parametric analyses. A *p*-value ≤ 0.05 was considered statistically significant. 

One-way between-groups multivariate analyses of variance were performed to identify the overall risk behaviors associated with the components of the personalized well-being index. These multivariate analyses were based on Wilks’ Lambda statistic after the Bonferroni adjustment of significance for multiple tests based on 14 risk behaviors tested. In case of significance in the multivariate analysis, post-hoc analyses were conducted for each significant risk behavior to explore inter-group differences for each component of PWBI.

## 3. Results

Association between Risk Behaviors and Personalized Well-Being Index in Study Population.

A total of 450 participants (32% girls, age: 19.4 ± 1.9) were included in the analyses. Table 1 and Table 2 show the frequency of risk behaviors in the total population. School dropout adolescents reported a PWBI of 61.81 ± 13.07, articulated into: social context (24.02 ± 5.25), emotional status (24.91 ± 6.89), and lifestyle habits (12.87 ± 2.80). Table 3 summarizes the results of multivariate analyses for associations between risk behaviors and the personalized well-being index (PWBI).

After an adjustment for multiple tests, PWBI was found to be significantly associated with eating disorders (Wilks’ Lambda = 0.89, adj. *p* < 0.001), abuse of psychotropic drugs (Wilks’ Lambda = 0.94 adj. *p* < 0.001), and frequency of drug use (Wilks’ Lambda = 0.92, adj. *p* < 0.05), whereas there were no significant differences for other risk behaviors (all adj. *p* > 0.05). Based on the significant associations that emerged between these selected risk behaviors and PWBI, more exhaustive post hoc analyses were conducted to assess which PWBI components contributed to these associations. With regard to eating disorders, they were associated with an impairment of the emotional status component (F = 39.64; *p* < 0.001) and a reduction in well-being as PWBI (F = 22.85; *p* < 0.001). An association was also found between psychotropic drugs use and all well-being components. In detail, the use of these drugs has a negative impact on social context, reducing interactions with peers, family, and school (F = 16.76; *p* < 0.001). Psychotropic drugs use/abuse also worsened emotional responses such as mood and self-perception (F = 22.74; *p* < 0.001) and lifestyle habits (F = 8.78; *p* < 0.01), and it compromised well-being perception (F = 23.31; *p* < 0.001).

The social context (F = 5.75; *p* < 0.01), emotional background (F = 7.63; *p* < 0.001), and global well-being perception (F = 7.70, *p* < 0.001) were negatively influenced by the frequency of psychotropic drugs use. 

Finally, social media addiction was related to an impairment of emotional responses (F = 11.77; *p* < 0.01) and cannabis use to a reduction in social relationships (F = 7.57; *p* < 0.05), while sexual activity (F = 6.33; *p* < 0.05) and contraceptive use (F = 7.12; *p* < 0.05) enhanced lifestyle habits.

## 4. Discussion

The present study describes the assessment of the PWBI in a sample of school dropout adolescents to evaluate if classical risk behaviors are capable of modifying well-being perception. The main findings can be summarized according to two points of view: (1) considering PWBI as a total score and (2) analyzing the individual components of the index. Firstly, among the different risk behaviors, those showing an association with well-being perception are eating disorders and psychotropic drugs use. In a next step, according to a more detailed statistical method for the quantification of associations between risk behaviors and single components of PWBI, the principal findings can be summarized as follows: (i) emotional status and well-being perception increase in the absence of eating disorders, psychotropic drugs use, and social media addiction; (ii) social context is better if cannabis and psychotropic drugs are not consumed; and (iii) lifestyle habits improve in the presence of sexual activity. 

To our knowledge, these findings, obtained on a sample of school dropout adolescents, are new because there are no data in the literature on the relationship between risk behavior and well-being perception in this category of the population. However, our data concerning the association between eating disorders and perceptions of well-being are in line with the findings obtained in adolescents, where among the risk factors attributable to the presence of eating disorders, social environment is the most important one [17,18]. In particular, the family environment seems to influence the shaping of eating habits and the evolution of disturbed behavior [19]. Evidence shows that family functioning, communication, and support are related to risk behaviors, mainly due to changing family dynamics [20,21]. Consequently, a poor family environment is linked to eating disorders, while, on the contrary, family cohesion is protective [22]. Although not directly, this may be in line with what we have shown in our data, according to which the social context is also involved; the stronger and more stable it is, the less risk behaviors are enacted [23]. In fact, although there are no data on adolescents dropping out of school, research on the general population suggests that not only does family play a pivotal role in preventing eating disorders, but there also seems to be a gender difference correlated with the frequency of family meals [23]. Family meals may promote a model of healthy eating behaviors and create a communication of cohesion, developing a healthier perception of self, and thus, accordingly to our approach, a better well-being perception [9,24]. On the contrary, negative family experiences that induce lower confidence are associated with an increase in eating disorders [25]. Another important result concerns the relationship between psychotropic drugs use and the well-being perception. Previous data have indicated that dropouts are characterized by an increased risk of substance use and other deviant behaviors [2]. However, in this population group, there is no definite data, other than retrospective evidences showing a correlation between dropout and a greater vulnerability in adulthood to delinquency and substances of abuse [26]. To our knowledge, only one study has been published describing the use of these substances among adolescent school dropouts, showing links between sociodemographic, interpersonal, cognitive, and personality characteristics and use rates [27]. Generally, there has been a gradual increase in the use of psychotropic drugs, stimulants, antidepressants, and antipsychotics in adolescents, greater in the USA than in Europe, probably due to the prevalence of nicotine and opioid addiction; however, the data are not complete because not all potential variables are considered [28]. In fact, our data show that the social context is also involved, improving in the case of non-substance taking, while, conversely, it is reduced under addiction conditions, with a compromised well-being perception. 

Finally, in accordance with previous data regarding lifestyle habits, a positive association with sexual activity has been reported. There is a link between unhealthy lifestyle factors and sexual dysfunction or inactivity. In particular, physical inactivity, alcohol consumption, tobacco smoking, and drug use appear to be associated with one or more sexual dysfunctions [29]. 

Furthermore, it is important to emphasize that most of the data in the literature where adolescents who have dropped out of school are mentioned refers to dropping out permanently. In our case, although they have dropped out of school, they are in a training agency for professional reintegration. This may explain why there is no clear prevalence of risk behaviors traditionally associated with this category, especially because of the healthy role of education. Several studies now identify school, learning, and education as protective factors against the onset of pathologies [8]. 

Moreover, an innovative aspect lies in the application of a multidimensional and personalized score for well-being perception evaluation. This index is based on the relationship between the different weights of the variables belonging to the three dimensions, lifestyle, emotional status, and social context, including subjective information on the state of health based on the individual perception of health, from social to psychological. The PWBI, already developed on a different class of adolescents (10–14 years) as described in Mastorci and colleagues, is here enriched with risk behaviors in order to understand how and if they interfere with well-being [9,30]. Our results, showing a relationship between the index and certain risk behaviors, in particular eating disorders and substance abuse, show how these deviant behaviors have a negative impact, not on individual dimensions, but on the integration between them. This is precisely the innovativeness of this score, which makes it possible to quantify how much the components related to health and well-being influence each other. 

Some limitations also warrant discussion. First, the sample consisted of 450 dropout adolescents between the ages of 18 and 21 living in North Italy, and it is therefore not representative of the Italian Country and the dropout phenomenon. Subsequent studies on a larger and more representative population will be necessary to confirm our preliminary results.

Moreover, the assessments were all based on self-report, and future work should include the use of instruments that provide more comprehensive sub-dimensions for previous experience as well as integrating information from school or, in this case, from the training agency where the young person is doing vocational training. Furthermore, the motivation for dropping out of school or any previous negative experiences were not assessed.

## 5. Conclusions

The PWBI does not focus on the identification of a disorder but rather on the relationship between the perception of well-being and all its constituent variables, including risk behaviors. Accordingly, we showed that well-being, as assessed with the PWBI, is influenced by risk behaviors such as eating disorders and psychotropic drug use. When we consider the individual components of the PWBI, eating disorders, psychotropic drug use, and social media addiction interfere with the emotional status and well-being perception, while cannabis and psychotropic drug use interfere with the social context, and sexual activity improves lifestyle habits. Thus, this study highlights the change of paradigm from a disease-oriented model to an educationally strength-based model when monitoring psychosocial well-being in order to define preventive and health promotion strategies in a more vulnerable category of the population. 

## Figures and Tables

**Table 1:** Frequency of substance use and abuse in total population.

Variables			Total (*n* = 450)
**Nicotine**		Yes	218 (48)
		Not	192 (43)
	Frequency (die)	0	172 (38)
		1–9	144 (32)
		10–30	69 (15)
		Over 40	2 (0)
**Cannabis**		Yes	76 (17)
		Not	320 (71)
	Frequency (last month)	0	294 (65)
		1–9	41 (9)
		10–30	18 (4)
		Over 40	14 (3)
**Illegal Drugs Use**		Yes	10 (2)
		Not	388 (86)
	Frequency (last month)	0	332 (74)
		1–9	7 (2)
		10–30	2 (0)
		Over 40	2 (0)
**Psychotropic Drugs**		Yes	22 (5)
		Not	376 (84)
	Frequency (last month)	0	332 (74)
		1–9	5 (1)
		10–30	10 (2)
		Over 40	3 (1)
**Alcoholic Drinks**		Yes	177 (39)
		Not	227 (50)
	Frequency (last month)	0	248 (55)
		1–9	121 (27)
		10–30	10 (2)
		Over 40	3 (1)

Data are expressed as number (%).

**Table 2:** Frequency of risk behaviors in total population.

Variables		Total (*n* = 450)
**Sexual Behavior**	Sexual activity	250 (56)
	Not Sexual activity	172 (38)
	Contraceptives use	211 (69)
	Not Contraceptives use	194 (43)
**BSMAS**	AUSM	6 (1)
	Not AUSM	376 (84)
**EAT-26**	ED risk	33 (7)
	ED no risk	364 (81)

Data are expressed as number (%). BSMAS: Bergen Social Media Addiction Scale; EAT-26: Eating Attitudes Test; AUSM: addictive use of social media; ED: eating disorders.

**Table 3:** Associations between risk behaviors and components of personalized well-being index (PWBI) assessed by multivariate analysis of variance.

Risk Behaviors	Wilks’ Lambda	F	Sig.	Adj. Sig.
Nicotine	0.99	1.65	0.18	1.00
Frequency (last month)	0.96	1.73	0.08	1.00
Cannabis	0.98	2.88	0.04	0.50
Frequency (last month)	0.94	2.29	0.02	022
Illegal drugs use	0.97	3.08	0.03	0.38
Frequency (last month)	0.94	2.12	0.03	0.36
Psychotropic drugs	0.94	8.25	<0.001	<0.001 *
Frequency (last month)	0.92	2.78	<0.01	<0.05 *
Alcoholic drinks	1.00	0.21	0.89	1.00
Frequency (last month)	0.96	1.50	0.14	1.00
Eating Disorders	0.89	15.08	<0.001	<0.001 *
Social Media Addiction	0.97	3.93	0.009	0.12
Sexual activity	0.98	3.15	0.02	0.35
Contraceptive use	0.98	2.72	0.04	0.62

*: adjusted *p*-value < 0.05 after Bonferroni correction of significance.

## Data Availability

The datasets used and/or analyzed during the current study are available from the corresponding author on reasonable request.
